# Enhancing functional production of a chaperone-dependent lipase in *Escherichia coli *using the dual expression cassette plasmid

**DOI:** 10.1186/1475-2859-11-29

**Published:** 2012-03-01

**Authors:** Thi Dinh Quyen, Chi Hai Vu, Giang Thi Thu Le

**Affiliations:** 1 Institute of Biotechnology, Vietnam Academy of Science and Technology, 18 Hoang Quoc Viet Road, Distr. Caugiay 10600, Hanoi, Vietnam

**Keywords:** *Ralstonia *sp. M1, Lipase, Chaperone, Functional expression, Secretion

## Abstract

**Abstracts:**

**Background**

The lipase subfamilies I.1 and I.2 show more than 33% homology in the amino acid sequences and most members share another common property that their genes are clustered with the secondary genes whose protein products are required for folding the lipase into an active conformation and secretion into the culture medium. In previous studies, the lipase (LipA) and its chaperone (LipB) from *Ralstonia *sp. M1 were overexpressed in *E. coli *and the lipase was successfully refolded *in vitro*. The purpose of this study was to enhance the production of the active lipase LipA from *Ralstonia *sp. M1 in the heterologous host *E. coli *without *in vitro *refolding process, using two-plasmid co-expression systems and dual expression cassette plasmid systems.

**Results**

To produce more active lipase from *Ralstonia *sp. M1 in *E. coli *without *in vitro *refolding process but with the help of overexpression of the chaperone (LipB1 and LipB3 corresponding to 56-aa truncated and 26-aa truncated chaperone LipB), six different expression systems including 2 two-plasmid co-expression systems (*E. coli *BL21/pELipAB^a ^+ pELipB1^k ^and BL21/pELipAB^a ^+ pELipB3^k^) and 4 dual expression cassette plasmid systems (BL21/pELipAB-LipB1^a^, BL21/pELipAB-LipB3^a^, BL21/pELipA-LipB1^a^, and BL21/pELipA-LipB3^a^) were constructed. The two-plasmid co-expression systems (*E. coli *BL21/pELipAB^a ^+ pELipB1^k ^and BL21/pELipAB^a ^+ pELipB3^k^) produced the active lipase at a level of 4 times as high as the single expression cassette plasmid system *E. coli *BL21/pELipAB^a ^did. For the first time, the dual expression cassette plasmid systems BL21/pELipAB-LipB1^a ^and BL21/pELipAB-LipB3^a ^yielded 29- and 19-fold production of the active lipase in comparison with the single expression cassette plasmid system *E. coli *BL21/pELipAB^a^, respectively. Although the lipase amount was equally expressed in all these expression systems (40% of total cellular protein) and only a small fraction of the overexpressed lipase was folded *in vivo *into the functional lipase in soluble form whereas the main fraction was still inactive in the form of inclusion bodies. Another controversial finding was that the dual expression cassette plasmid systems *E. coli *BL21/pELipAB-LipB1^a ^and *E. coli*/pELipAB-LipB3^a ^secreted the active lipase into the culture medium of 51 and 29 times as high as the single expression cassette plasmid system *E. coli *pELipAB^a ^did, respectively, which has never been reported before. Another interesting finding was that the lipase form LipA6xHis (mature lipase fused with 6× histidine tag) expressed in the dual expression cassette plasmid systems (BL21/pELipA-LipB1^a ^and BL21/pELipA-LipB3^a^) showed no lipase activity although the expression level of the lipase and two chaperone forms LipB1 and LipB3 in these systems remained as high as that in *E. coli *BL21/pELipAB^a ^+ pELipB1^k^, BL21/pELipAB^a ^+ pELipB3^k^, BL21/pELipAB-LipB1^a^, and BL21/pELipAB-LipB3^a^. The addition of Neptune oil or detergents into the LB medium increased the lipase production and secretion by up to 94%.

**Conclusions**

Our findings demonstrated that a dual expression cassette plasmid system *E. coli *could overproduce and secrete the active chaperone-dependent lipase (subfamilies I.1 and I.2) *in vivo *and an improved dual expression cassette plasmid system *E. coli *could be potentially applied for industrial-scale production of subfamily I.1 and I.2 lipases.

## Background

Lipases (triacylglycerol ester hydrolases, EC 3.1.1.3) catalyze the hydrolysis of long-chain triacylglycerols in aqueous media to liberate diacyl-, monoacylglycerol, glycerol and fatty acids [1]. In non-aqueous media, they catalyze the esterification and transesterification reactions [2]. Unlike esterases, lipases prefer long chain fatty acids and are activated in the interface of lipid substrate and water. Owing to their substrate specificity, region specificity, chiral selectivity, thermostability, and alkaline stability, microbial lipases have been utilized widely in industrial fields for the production or modification of detergents, dairy products and diagnostics, as well as for oil processing, biotransformations, and chiral separation or synthesis [3].

Bacterial lipase family I has been divided into seven subfamilies on basis of amino acid sequence homology [3]. Subfamily I.1 contains the lipases from *Pseudomonas aeruginosa *[4,5], *Pseudomonas fragi *[6], and *Acinetobacter *sp. [7,8], and subfamily I.2 comprises the lipases from *Pseudomonas glumae *[9], *Pseudomonas luteola *[10], *Burkholderia cepacia *[11], *Chromobacterium viscosum *[12], and *Ralstonia *sp. M1 [13].

These two lipase subfamilies I.1 and I.2 show more than 33% homology in the amino acid sequences [14] and most members share another common property that their genes are clustered with the secondary genes located immediately downstream [4,5,8,11,13,15] or upstream of the lipase genes [7]. Protein products of the secondary genes are so-called lipase-specific foldases (modulator, activator, helper protein or chaperone) required for folding the lipase into an active conformation and secretion into the culture medium. During the secretion process of the lipase via the two-step type II secretion pathway, the lipase chaperones directly participate in the *in vivo *folding and secretion process.

In spite of various applications of *Pseudomonas *and *Burkholderia *lipases from subfamilies I.1 and I.2, respectively, as detergent additive and for variety of biotransformations, overproduction of the functional lipase in *E. coli *has been much difficult due to the requirement of the chaperone to fold the lipase into an active conformation [14]. To enhance the functional expression of the lipases in *E. coli*, two strategies have been developed. The first one was to produce and purify both the lipase and its chaperone separately in *E. coli *and the fully active lipase was subsequently achieved by *in vitro *refolding process using various approaches [11,16-20]. Recently, the second strategy was directed to produce *in vivo *functional lipases in heterologous host *E. coli *using two-plasmid co-expression system without the need of *in vitro *refolding [21-24]. To date, expression of *in vivo *functional lipase in heterologous host *E. coli *using a dual expression cassette plasmid system has not been achieved.

In the previous study [13], the lipase-encoding gene *lipA *and its chaperone-encoding gene *lipB *from the *Ralstonia *sp. strain M1 were cloned, sequenced, and overexpressed in *E. coli *BL21 separately. The recombinant lipase was an alkaline, thermophilic, highly organic-solvent-resistant and detergent-inducible lipase. The plasmid pELipAB^a ^carrying the gene cluster encoding LipA and LipB under the control of T7 promoter was expressed in *E. coli *strain BL21. The amount of LipA accounted for 40% total cellular protein, however the amount of LipB was just less than 1% in comparison with the total amount of proteins. On the other hand, for the optimum *in vitro *refolding of the lipase, both LipA and LipB are required in equal amounts to form the lipase:chaperone (1:1) complex [19]. That means the unequal expression level of the lipase and its chaperone in the heterologous host led to the low formation of the active lipase *in vivo*, only 1-3% of the expressed lipase were *in vivo *activated.

To enhance the amount of the functional lipase, we overexpressed LipA (70 mg per gram cell wet weight) and LipB1 (12 mg per gram cell wet weight) in *E. coli *BL21 and the *in vitro *refolding increased the lipase activity by 4.4 times. However, in these studies, *in vitro *refolding was required to fold the lipase into active conformation. The purpose of the present study was to enhance the production of the active lipase LipA from *Ralstonia *sp. M1 in the heterologous host *E. coli *without *in vitro *refolding process, using two-plasmid co-expression systems and dual expression cassette plasmid systems. For the first time, an active lipase was expressed in a dual expression cassette plasmid system *E. coli*.

## Results and discussions

### Functional expression of lipase using two-plasmid co-expression system

To express the functional lipase in *E. coli *without *in vitro *refolding process, the first strategy was developed to enhance the expression level of the chaperone in *E. coli *using two-plasmid co-expression systems *E. coli *BL21/pELipAB^a ^+ pELipB1^k ^and BL21/pELipAB^a ^+ pELipB3^k^.

#### Expression level of LipA and LipB

*E. coli *BL21/pELipAB^a ^+ pELipB1^k ^and BL21/pELipAB^a ^+ pELipB3^k ^were cultivated and induced by IPTG for the production of the lipase and chaperone LipA + LipB1 and LipA + LipB3, respectively. In the previous study, the active lipase was negligible when the co-expressed chaperone LipB in the gene cluster was expressed at a negligible level in comparison to the lipase LipA using plasmid pELipAB^a ^[25]. Thus, in this study, we tried to enhance the expression level of the chaperone in the form of LipB1 or LipB3 to produce more active lipase molecules *in vivo*.

**Figure 1:**
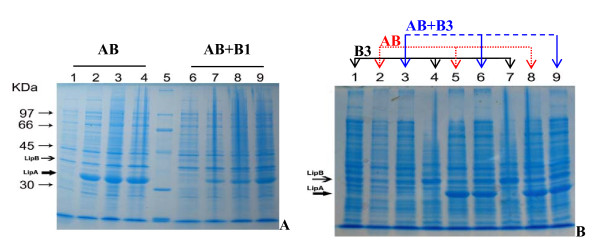
**SDS-PAGE of BL21 cell lysates using two-plasmid co-expression system**. (**A)**. pELipAB^a ^(1-4), pELipAB^a ^+ pELipB1^k ^(6-9). Lanes (1-4) and (6-9) indicated induction by IPTG for 0, 1, 2, and 4 hours, respectively; Lane 5: standard protein. **(B)**. pELipB3^k ^(1, 4, 7), pELipAB^a ^(2, 5, 8) and pELipAB^a ^+ pELipB3^k ^(3, 6, 9). Lanes (1, 2, 3), (4, 5, 6), (7, 8, 9) indicated induction by IPTG for 0, 2, and 4 hours, respectively.

The expression level of LipA in BL21/pELipAB^a ^+ pELipB1^k ^(10-21% of total cellular protein, using densitometry analysis Dolphin 1-D software operation) (Figure 1A, lane 7-9) was quite lower than that in BL21/pELipAB^a ^(30-40%) (Figure 1A, lane 2-4). The expression level of LipA in BL21/pELipAB^a ^after induction by IPTG for 1 hour (40%) (Figure 1A, lane 2) was higher than that in BL21/pELipAB^a ^+ pELipB1^k ^after induction by IPTG for 4 hours (21%) (Figure 1A, lane 9). However, the amount of chaperone LipB (Figure 1A, lane 2-4) and LipB + LipB3 (Figure 1A, lane 7-9) expressed in two systems BL21/pELipAB^a ^and BL21/pELipAB^a ^+ pELipB1^k^, respectively was also not observed by SDS-PAGE even after induction for 4 hours.

The lipase LipA was expressed in BL21/pELipAB^a ^(Figure 1B, lane 2, 5, 8) and BL21/pELipAB^a ^+ pELipB3^k ^(Figure 1B, lane 3, 6, 9) in an equal level (25%) and obviously observed, whereas the expression level of the protein chaperones LipB3 (in BL21/pELipB3^k^, Figure 1B, lane 1, 4, 7), LipB (in BL21/pELipAB^a^, Figure 1B, lane 2, 5, 8) and LipB + LipB3 (BL21/pELipAB^a ^+ pELipB3^k^, Figure 1B, lane 3, 6, 9) was not observed by SDS-PAGE even after induction by IPTG for 4 hours.

#### Production of functional lipase

The lipase assay was performed with olive oil as substrate by a continuous titration method. The two-plasmid co-expression systems *E. coli *BL21/pELipAB^a ^+ pELipB1^k ^and BL21/pELipAB^a ^+ pELipB3^k ^produced the functional lipase of 493 U/g and 440 U/g cell wet weight corresponding to 4.6 and 4.1 times as high as the single-plasmid expression system BL21/pELipAB^a ^(107 U/g) did, respectively (Table 1) although the expression level of LipA in *E. coli *BL21/pELipAB^a ^+ pELipB1^k ^and BL21/pELipAB^a ^+ pELipB3^k ^(21-25%) was lower than that of LipA in BL21/pELipAB^a ^(25-40%). The only explanation was that the two-plasmid co-expression systems BL21/pELipAB^a ^+ pELipB1^k ^and BL21/pELipAB^a ^+ pELipB3^k ^expressed more chaperone molecules (LipB + LipB1 and LipB + LipB3, respectively) than the single plasmid expression system BL21/pELipAB^a ^(only LipB) and this led to approximately 4-5 times *in vivo *lipase folding in both the two-plasmid co-expression systems.

**Table 1:** Production of the active lipase LipA by the two-plasmid co-expression systems BL21/pELipAB^a ^+ pELipB1^k ^and BL21/pELipAB^a ^+ pELipB3^k^

Expression system BL21/	Whole cell lysate	
	
	U/g	%
pELipAB^a^	107.3 ± 3.6	100 ± 3.4
pELipAB^a ^+ pELipB1^k^	492.7 ± 3.1	458.9 ± 2.9
pELipAB^a ^+ pELipB3^k^	440.3 ± 5.7	410.2 ± 5.3

The result of *in vivo *folding was very similar to *in vitro *refolding in the previous study [13]. *In vitro *refolding of the lipase LipA expressed in the single plasmid expression system BL21/pELipAB^a ^using the optimal ratio of lipase to chaperone for refolding (1:10) led to the optimal formation of the functional lipase (650 U/mg lipase protein), 4 times as high as the functional lipase LipA without *in vitro *refolding (151 U/mg). Recently, *in vivo *expression of functional *Pseudomonas *and *Burkholderia *lipases of the subfamilies I.1 and I.2 were achieved by two-plasmid co-expression system *E. coli *BL21(DE3) [21-23]. However, no direct comparison between *in vitro *refolding and *in vivo *folding efficacy was reported so far in these studies.

### Functional expression of lipase using dual expression cassette plasmid system

The second strategy was directed to enhance the expression level of the chaperone in *E. coli *and thus to enhance the production of the functional lipase without *in vitro *refolding process using the dual expression cassette plasmid systems *E. coli *BL21/pELipAB-LipB1^a ^and pELipAB-LipB3^a^.

#### Expression level of LipA and LipB

**Figure 2:**
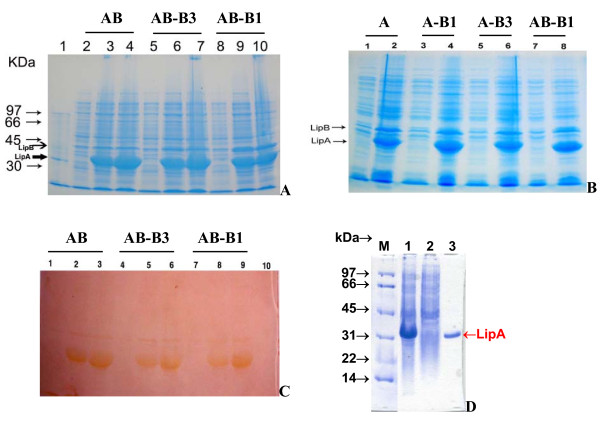
**SDS-PAGE of BL21 cell lysates using dual expression cassette plasmid system**. **(A)**. pELipAB^a ^(2-4), pELipAB-LipB3^a ^(5-7), pELipAB-LipB1^a ^(8-10). Lanes (2, 5, 8), (3, 6, 9), (4, 7, 10) indicated induction by IPTG for 0, 2, and 4 hours, respectively; Lane 1: standard protein. **(B)**. pELipA^a ^(1-2), pELipA-LipB1^a ^(3-4), pELipA-LipB3^a ^(5-6), pELipAB-LipB1^a ^(7-8). Odd and even lanes indicated induction by IPTG for 0 and 4 hours, respectively. **(C)**. Lipase-activity stained SDS-PAGE of BL21 cell lysate harboring the recombinant plasmids: pELipAB^a ^(1-3), pELipAB-LipB3^a ^(4-6), pELipAB-LipB1^a ^(7-9). Lanes (1, 4, 7), (2, 5, 8), and (3, 6, 9) indicated induction by IPTG for 0, 2, and 4 hours, respectively. **(D)**. pELipAB-LipB1^a ^whole cell lysate (1), cell lysate's supernatant (2), cell lysate precipitate (3).

The expression level of the lipase LipA (Figure 2) was relatively equal (~40%) in all dual expression cassette plasmid systems *E. coli *BL21/pELipAB-LipB1^a ^(Figure 2A, lane 10, B lane 8), BL21/pELipAB-LipB3^a ^(Figure 2A, lane 7), BL21/pELipA-LipB1^a ^(Figure 2B, lane 4), BL21/pELipA-LipB3^a ^(Figure 2B, lane 6) as well as in the single expression cassette plasmid systems BL21/pELipAB^a ^(Figure 2A, lane 4) and BL21/pELipA^a ^(Figure 2B, lane 2). The lipase activity staining also confirmed that the amount of the lipase LipA was produced equally in the dual expression cassette plasmid systems *E. coli *BL21/pELipAB-LipB1^a ^(Figure 2C, lane 9), BL21/pELipAB-LipB3^a ^(Figure 2C, lane 6), and BL21/pELipAB^a ^(Figure 2C, lane 3).

Similar to the two-plasmid co-expression systems, the protein chaperone LipB, LipB1 and LipB3 expressed in the dual expression cassette systems *E. coli *BL21/pELipAB-LipB1^a ^(Figure 2A, lane 10, B lane 8), BL21/pELipAB-LipB3^a ^(Figure 2A, lane 7), BL21/pELipA-LipB1^a ^(Figure 2B, lane 4), BL21/pELipA-LipB3^a ^(Figure 2B, lane 6) and in the single expression cassette plasmid system BL21/pELipAB^a ^(Figure 2A, lane 4) was difficult to be detected by SDS-PAGE.

#### Production of functional lipase

After induction by 1 mM IPTG for 4 hours, the dual expression cassette plasmid systems *E. coli *BL21/pELipAB-LipB1^a ^and pELipAB-LipB3^a ^produced the functional lipase at the level of 6473 U/g and 4282 U/g cell wet weight corresponding to 28.5 and 18.9 times as high as the single expression cassette plasmid system BL21/pELipAB^a ^(227 U/g cell wet weight) did, respectively (Table 2). The dual expression cassette plasmid systems *E. coli *BL21/pELipAB-LipB1^a ^and pELipAB-LipB3^a ^secreted the functional lipase at the level of 5065 and 2930 U/g cell wet weight, corresponding to 50.7 and 29.3 times as high as the single expression cassette plasmid system BL21/pELipAB^a ^(100 U/g cell wet weight) did, respectively.

**Table 2:** Production of the active lipase LipA by the dual expression cassette plasmid systems BL21/pELipAB-LipB1^a ^and BL21/pELipAB-LipB3^a^

Expression system BL21/	Culture supernatant		Whole cell lysate		Whole culture		Cell lysate's supernatant		Cell lysate's precipitate	
	
	U/g	%	U/g	%	U/g	%	U/g	%	U/g	%
pELipAB^a^	99.9 ± 1.5	100 ± 1.5	127.5 ± 1.1	100 ± 0.8	227.4 ± 0.9	100 ± 0.4	32.4 ± 1.6	21.3 ± 1.1	119.7 ± 8.6	78.7 ± 5.6
pELipAB-LipB1^a^	5064.7 ± 11.2	5068.4 ± 11.2	1408.3 ± 6.9	1104.5 ± 5.4	6473.0 ± 18.0	2846.1 ± 7.9	267.9 ± 9.6	17.2 ± 0.6	1287.9 ± 49.4	82.8 ± 3.2
pELipAB-LipB3^a^	2930.8 ± 4.5	2932.9 ± 4.5	1352.1 ± 4.2	1060.4 ± 3.3	4282.9 ± 8.5	1883.1 ± 3.7	192.0 ± 7.0	11.8 ± 0.4	1437.9 ± 47.1	88.2 ± 2.9

Although the expression level of the chaperone (LipB + LipB1 and LipB + LipB3) in the dual expression cassette plasmid systems was not able to be observed on SDS-PAGE and the expression level of the lipase LipA was relatively equal (~40% of total cellular protein), the production of the functional lipase was increased to 29 and 19 times, respectively. The explanation might be that the number of the chaperone molecules (LipB + LipB1 and LipB + LipB3) expressed in the dual expression cassette plasmid system was higher than that (only LipB) in the single expression cassette plasmid system. In the sequence, it led to enhance the number of *in vivo *folded lipase molecules. In our previous study [26], we also expressed the lipase from *Burkholderia cepacia *ATCC 21808 in heterologous host *E. coli *using a dual expression cassette plasmid with the pre-signal peptide ompA. The functional lipase expressed in the dual expression cassette plasmid system increased to 450 folds (18000 U/g) in comparison with that expressed in the single expression cassette plasmid system (40 U/g), although the lipase level decreased from 40% to 15% of the total proteins [26] due to simultaneous overexpression of the chaperone. To our knowledge, there has been no report on the expression of the functional (*Pseudomonas *and *Burkholderia*, subfamily I.1 and I.2) lipase using a dual expression cassette system.

The functional lipase in the culture supernatant of the dual expression cassette plasmid systems *E. coli *BL21/pELipAB-LipB1^a ^and pELipAB-LipB3^a ^was 50.7 and 29.3 times as high as the functional lipase in the culture supernatant of the single expression cassette plasmid system *E. coli *BL21/pELipAB^a^. This finding was somehow controversial to other reports. Peng *et al. *reported that no lipase activity was detected in the culture supernatant of the recombinant *E. coli *BL21(DE3) co-expressing the lipase and its chaperone from *Pseudomonas aeruginosa *CS-2 [23]. The lipase activity was also not detected in the culture supernatant by the recombinant *E. coli *JM109 harboring a plasmid containing both lipase and chaperone gene from *P. aeruginosa *LST-03 [27]. The secretion of subfamily I.1 and I.2 lipases did not proceed properly in heterologous hosts [28].

#### Functional lipase in soluble form

One question was raised where was the functional (*in vivo *folded) lipase present in the soluble form or in form of inclusion bodies? We have expressed the lipase LipA in the dual expression cassette plasmid systems *E. coli *BL21/pELipAB-LipB1^a ^and BL21/pELipAB-LipB3^a ^and the single expression cassette plasmid system BL21/pELipAB1^a^. The cell pellets were sonified and the cell lysates were centrifuged to separate the cell lysate's supernatant (soluble fraction) and the cell lysate's precipitate (insoluble fraction). The lipase molecules LipA were retained most in the cell lysate's precipitate as inclusion bodies (Figure 2D, lane 3) and not observed in the cell lysate's supernatant as soluble form (Figure 2D, lane 2). However, the surprising finding was that the cell lysate's supernatant contained 88, 83, and 79% of the total functional lipase production in the dual expression cassette plasmid systems *E. coli *BL21/pELipAB-LipB1^a ^and BL21/pELipAB-LipB3^a ^and the single expression cassette plasmid system BL21/pELipAB, respectively, whereas the cell lysate's precipitate showed only 12, 17 and 21% of the total functional lipase production, respectively. This demonstrated that only a small fraction of the expressed lipase was folded *in vivo *and become active in soluble form whereas the major fraction of the expressed lipase was still not folded *in vivo *and thus maintained inactive as inclusion bodies. This study suggested that it is still a big potential to enhance functional production of the chaperone-dependent lipase.

### Non-functional expression of lipase using dual expression cassette plasmid system

The expression level of the lipase expressed in the dual expression cassette plasmid systems *E. coli *BL21/pELipA-LipB1^a ^(Figure 2B, lane 4) and BL21/pELipA-LipB3^a ^(Figure 2B, lane 6) was relatively equal (~40% of total cellular protein) to that in BL21/pELipAB-LipB1^a ^(Figure 2A, lane 10), BL21/pELipAB-LipB3^a ^(Figure 2A, lane 7), as well as in the single expression cassette plasmid system BL21/pELipA^a ^(Figure 2B, lane 2), and BL21/pELipAB^a ^(Figure 2A, lane 4). However, no lipase activity was detected in the cell culture supernatant as well as in the cell lysate of the dual expression cassette plasmid systems BL21/pELipA-LipB1^a ^and BL21/pELipA-LipB3^a^. The expression level of the chaperone B1 and B3 in the dual expression cassette plasmid systems BL21/pELipA-LipB1^a ^(Figure 2B, lane 4) and BL21/pELipA-LipB3^a ^(Figure 2B, lane 6) was relatively equal to that in BL21/pELipAB-LipB1^a ^(Figure 2A, lane 10) and BL21/pELipAB-LipB3^a ^(Figure 2A, lane 7). But why did BL21/pELipA-LipB1^a ^and BL21/pELipA-LipB3^a ^form no active lipase? The reason might be that the lipase molecule expressed in these systems was LipA fused with the 6x histidine tag, so that the structure of the lipase molecule might be changed and led to be not properly folded *in vivo *and no active lipase was found. Although the level of the lipase and the chaperone expressed in these systems (Figure 2B) was quite similar to those in BL21/pELipAB-LipB1^a ^and BL21/pELipAB-LipB3^a ^(Figure 2A).

### Effect of culture components on production of active lipase by dual expression cassette plasmid systems

**Figure 3:**
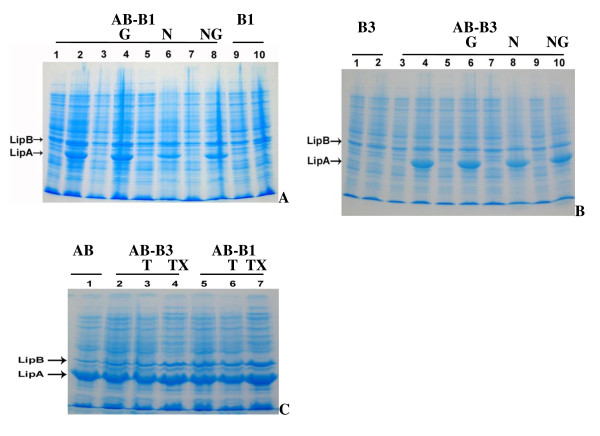
**SDS-PAGE of BL21 cell lysates harboring the recombinant plasmids**. **(A)**. pELipAB-LipB1^a ^(1-8) and pELipB1^k ^(9-10). LB medium without addititive (1-2), with 0.5% (v/v) of Neptune oil (5-6), 0.5% (w/v) of gum Arabic (3-4), 0.5% (w/v) of gum Arabic + 0.5% (v/v) of Neptune oil (7-8). Odd and even lanes indicated induction by IPTG for 0 and 4 hours, respectively. **(B)**. pELipB3^k ^(1-2) and pELipAB-LipB3^a ^(3-10). LB medium without additive (3-4), with 0.5% (w/v) of gum Arabic (5-6), 0.5% (v/v) of Neptune oil (7-8), 0.5% (w/v) of gum Arabic + 0.5% (v/v) of Neptune oil (9-10). Odd and even lanes indicated induction by IPTG for 0 and 4 hours, respectively. **(C)**. pELipAB^a ^(1); pELipAB-LipB3^a ^(2-4), pELipAB-LipB1^a ^(5-7). LB medium without additive (1, 2, 5), with 0.5% of Tween 80 (3, 6), and 0.5% of Triton X-100 (4, 7) after induction by IPTG for 4 hours.

To figure out the effect of some additives including Neptune oil, gum Arabic, Tween 80, and Triton X-100 on the formation of the active lipase *in vivo *in the dual expression cassette plasmid systems BL21/pELipAB-LipB1^a ^and pELipAB-LipB3^a^, we carried out the expression of the recombinant *E. coli *cells in LB medium by adding additives as showed in Table 3. The addition of the substrate Neptune oil and gum Arabic into the culture media did not enhance the expression level of the lipase LipA in the dual expression cassette plasmid system BL21/pELipAB-LipB1^a ^(~30% of total cellular protein) (Figure 3A). The addition of Neptune oil decreased significantly the expression level of the lipase LipA (20-25% of total cellular protein) (Figure 3A, lane 6, 8). Gum Arabic (Figure 3A, lane 4), Tween 80 (Figure 3C, lane 6), and Triton X-100 (Figure 3C, lane 7) showed no different effect on the expression level of the lipase LipA (~30% of total cellular protein). The addition of 0.5% of Neptune oil plus 0.5% of gum Arabic showed a slight decrease of 2-10% in production of the active lipase by BL21/pELipAB-LipB1^a^, but the addition of 0.5% of Neptune oil plus 0.5% of gum Arabic, Tween 80 and Triton X-100 increased the active lipase production by 5-27% (Table 3).

**Table 3:** Effect of 0.5% additives on the production level of the functional lipase LipA by the dual expression cassette plasmid systems BL21/pELipAB-LipB3^a ^and BL21/pELipAB-LipB1^a^

Additive (0.5%) supplemented to LB medium	BL21/pELipAB-LipB1^a^-						BL21/pELipAB-LipB3^a^					
	
	Culture supernatant		Whole cell lysate		Whole culture		Culture supernatant		Whole cell lysate		Whole culture	
	
	U/g	%	U/g	%	U/g	%	U/g	%	U/g	%	U/g	%
LB medium	5064.7 ± 11.2	100.0 ± 0.2	1408.3 ± 6.9	100.0 ± 0.5	6473.0 ± 18.0	100.0 ± 0.3	2930.8 ± 4.5	100 ± 0.2	1352.1 ± 4.2	100.0 ± 0.3	4282.9 ± 8.5	100.0 ± 0.2
Neptune oil	5520.9 ± 104.4	109.0 ± 2.1	831.3 ± 39.6	59.0 ± 2.8	6352.2 ± 85.9	98.1 ± 1.3	5683.9 ± 51.6	193.9 ± 1.8	2636.3 ± 39.3	195.0 ± 2.9	8320.2 ± 61.2	194.3 ± 1.4
Gum Arabic	5268.0 ± 121.0	104.0 ± 2.4	549.3 ± 62.0	39.0 ± 4.4	5817.3 ± 170.5	89.9 ± 2.6	3926.4 ± 20.9	134.0 ± 0.7	1149.3 ± 18.5	85.0 ± 1.4	5075.6 ± 39.3	118.5 ± 0.9
Gum Arabic + Neptune oil	5420.0 ± 85.9	107.0 ± 1.7	1366.1 ± 51.7	97.0 ± 3.7	6786.1 ± 95.4	104.8 ± 1.5	4249.5 ± 46.8	145.0 ± 1.6	1743.7 ± 43.2	129.0 ± 3.2	5993.1 ± 81.9	139.9 ± 1.9
Tween 80	6179.2 ± 119.1	122.0 ± 2.4	1534.9 ± 55.2	109.0 ± 3.9	7714.0 ± 171.4	119.2 ± 2.6	3780.0 ± 70.1	129.0 ± 2.4	1014.5 ± 29.9	75.0 ± 2.2	4794.5 ± 57.6	111.9 ± 1.3
Triton X-100	8003.5 ± 72.0	158.0 ± 1.4	225.5 ± 34.5	16.0 ± 2.5	8229.0 ± 74.3	127.1 ± 1.1	4864.5 ± 67.2	166.0 ± 2.3	122.8 ± 12.4	9.1 ± 0.9	4987.3 ± 72.5	116.4 ± 1.7

In the dual expression cassette plasmid system BL21/pELipAB-LipB3^a^, the addition of Neptune oil (Figure 3B, lane 8), Neptune oil plus gum Arabic (Figure 3B, lane 10), gum Arabic (Figure 3B, lane 6), Tween 80 (Figure 3C, lane 3), Triton X-100 (Figure 3C, lane 4) showed no effect on the expression level of the lipase LipA (~30% of total cellular protein). However, the addition of 0.5% of Neptune oil and 0.5% of Neptune oil plus 0.5% of gum Arabic increased the production of the active lipase by the dual expression cassette plasmid system BL21/pELipAB-LipB3^a ^by 94 and 40%, respectively. Whereas the addition of 0.5% of gum Arabic, Tween 80, and Triton X-100 increased only by 12-16% (Table 3). The addition of Triton X-100, Tween 80, and gum Arabic could solubilize and activate the membrane-bound lipase, so that it could help the cell to secrete more lipase.

## Conclusions

The production of the active chaperone-dependent lipase from *Ralstonia *sp. M1 (lipase subfamily I.1) in heterologous host *E. coli *was enhanced up to 4 times by using two-plasmid co-expression systems and up to 19-29 times by using dual expression cassette plasmid systems in comparison to the single expression cassette plasmid systems. In dual expression cassette plasmid systems, the active lipase was secreted into the culture medium at the level of up to 29-51 times as high as the single expression cassette plasmid systems. Only a small fraction of the overexpressed lipase was *in vivo *folded into the functional lipase in soluble and secreted form and the main part was still inactive in form of inclusion bodies. The addition of Neptune oil, gum Arabic, Tween 80 and Triton X-100 led to double production and secretion of the active lipase in the dual expression cassette plasmid system *E. coli*. Our findings demonstrated that the dual expression cassette plasmid system *E. coli *could overproduce and secreted the active chaperone-dependent lipase (subfamilies I.1 and I.2) *in vivo *and an improved dual expression cassette plasmid system *E. coli *could be potentially applied for industrial-scale production of subfamily I.1 and I.2 lipases.

## Materials and methods

### Chemicals and reagents

Fast red TR, α-naphthyl acetate, tributyrin, olive oil, Triton X-100, Tween 80, and gum Arabic were purchased from Sigma-Aldrich Co. (St. Louis, USA). Bacto-tryptone and yeast extract were from Difco (Lawrence, USA). Restriction enzymes, CIAP, T4 ligase, and *Taq *polymerase were supplied by Fermentas (Thermo Fisher Scientific Inc., Waltham, USA). PCR mastermix was from Invitrogen Corp. (Carlsbad, USA). DNA Gel-Extraction Kit was from Qiagen (Venlo, Netherlands). All other reagents were of analytical grade unless otherwise stated.

### Plasmids, bacterial strains and culture conditions

*Escherichia coli *DH5α (*F^-^*, ø80d*lacZ*ΔM15, Δ(*lacZYA-argF*)U169, *deo*R, *recA*1, *endA*1, *hsdR*17(rk-, mk+), *phoA, supE*44, λ-, *thi*-1, *gyrA*96, *relA*1) and pCR2.1 TOPO (TOPO^® ^TA cloning Kit, Invitrogen Corp., Carlsbad, USA) were used to subclone the lipase gene *lipA *and chaperone gene *lipB*. The vectors pET22b(+) and pET28a(+) (Novagen, Merck KGaA, Darmstadt, Germany) and *Escherichia coli *BL21 (DE3) cells (*F^- ^ompT gal dcm lon hsdS_B_*(*r_B_^- ^m_B_^-^*) *λ*(*DE3 *[*lacI lacUV5-T7 gene 1 ind1 sam7 nin5*]) were used to express the lipase gene *lipA *and its chaperone gene *lipB *under the control of the T7-promoter induced by IPTG. LB medium (Luria-Bertani) containing 1% (w/v) bacto tryptone; 0.5% (w/v) yeast extract; 1% (w/v) NaCl; pH 7-7.5 was used for cultivation of *E. coli *DH5α and BL21 (DE3). LB agar contained additionally 2% (w/v) agar and 100 μg ampicillin/ml. Plasmids pELipAB^a^, pELipA^a^, pELipB1^a^, and pELipB3^a ^[25] were used to provide the lipase gene *lipA *and chaperone gene *lipB*. The plasmids used in this study are summarized in Table 4.

**Table 4:** Plasmids used in this work

Plasmid	Gene of interest	Reference
pET22b(+)	T7-promoter, Amp^r^, *pelB*, T7-terminator	Novagen, Merck KGaA, Darmstadt, Germany
pET28a(+)	T7-promoter, Kan^r^, T7-terminator	Novagen, Merck KGaA, Darmstadt, Germany
pCR2.1-TOPO	TOPO binding site, Amp^r^	Invitrogen Corp., Carlsbad, USA
pELipAB^a^	*lipA, lipB*, T7-promoter, Amp^r^, *pelB*, T7-terminator	[25]
pELipA^a^	*lipA*, T7-promoter, Amp^r^, *pelB*, T7-terminator	[25]
pELipB1^a^	*lipB1*, T7-promoter, Amp^r^, *pelB*, T7-terminator	[25]
pELipB3^a^	*lipB3*, T7-promoter, Amp^r^, *pelB*, T7-terminator	[25]
pELipAB^a^-LipB1^a^	*lipA, lipB, lipB1*, T7-promoter, Amp^r^, *pelB*, T7-terminator	In this study
pELipAB^a^-LipB3^a^	*lipA, lipB, lipB3*, T7-promoter, Amp^r^, *pelB*, T7-terminator	In this study
pELipA^a^-LipB1^a^	*lipA, lipB1*, T7-promoter, Amp^r^, *pelB*, T7-terminator	In this study
pELipA^a^-LipB3^a^	*lipA, lipB3*, T7-promoter, Amp^r^, *pelB*, T7-terminator	In this study
pELipB1^k^	*lipB1*, T7-promoter, Kan^r^, T7-terminator	In this study
pELipB3^k^	*lipB3*, T7-promoter, Kan^r^, T7-terminator	In this study

Amp^r^, ampicillin resistant; Kan^r^, kanamycin resistant; pelB, signal peptide; *lipAB*, gene cluster encoding LipA and LipB; *lipA*, gene encoding LipA; *lipB1*, gene encoding LipB1; *lipB3*, gene encoding LipB3.

### DNA manipulations

Plasmid DNA isolation was carried out by the method as previously described [29]. DNA fragments and PCR products were excised from a 0.8% agarose gel and purified by a gel extraction kit (Qiagen, Venlo, Netherlands) according to the manufacturer's instructions. DNA sequencing was performed on an ABI PRISM 3100 Avant Genetic Analyzer (Applied Biosystems Inc., Foster City, USA). *E. coli *DH5α and BL21 were transformed using heat shock methods as previously described [29].

### Construction of plasmids pELipB1^k ^and pELipB3^k^

**Figure 4:**
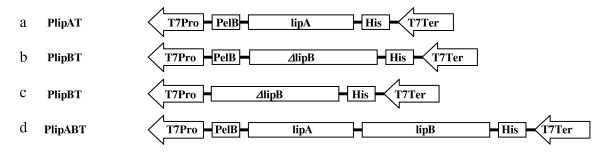
**Schematic representation of the expression cassettes in pELipA^a ^(a), pELipB1^a ^and pELipB3^a ^(b), pELipB1^k ^and pELipB3^k ^(c), pELipAB^a ^(d), pELipA-B1^a ^and pELipA-B3^a ^(a,b), pELipAB-B1^a ^and pELipAB-LipB3^a ^(d,b), pLipAB^a ^+ pELipB1^k ^and pLipAB^a ^+ pELipB1^k ^(d,c), derived from vector pET22b(+) and pET28a(+) for *E. coli *BL21**. T7Pro, T7 promoter; PelB, PelB signal peptide; *lipA*, mature lipase gene; His, 6xHis tag; T7Ter, T7 terminator; *ΔlipB*, truncated *lipB *ORF encoding a LipB derivative lacking the first 56 or 26 amino acids, corresponding to LipB1 and LipB3; *lipB*, the complete chaperone gene.

To co-express the lipase and chaperone using two different expression plasmids, two forms of the gene encoding chaperone LipB1 (N-terminal 56-aa truncated) and LipB3 (N-terminal 26-aa truncated) were isolated from the plasmid pELipB1^a ^and pELipB3^a^, respectively, by using the restriction enzymes *Nco*I and *Hin*dIII. The gene fragments *lipB1 *and *lipB3 *were inserted into the expression vector pET28a(+) also digested with *Nco*I and *Hin*dIII resulting in expression plasmids pELipB1^k ^and pELipB3^k^, respectively (Figure 4).

### Construction of plasmids pELipAB-LipB1^a ^and pELipAB-LipB3^a^

To construct plasmids harboring two expression cassettes promoter-gene-terminator, the expression plasmid pELipAB^a ^containing the expression cassette *PlipABT *(P: T7 promoter, lipase-chaperone gene cluster *lipAB*, T: T7 terminator, Figure 4) was used. The second expression cassette *PlipB1T *and *PlipB3T *(Figure 4) were amplified from the templates pELipB1^a ^and pELipB3^a^, respectively. The designed specific primers PEF (5'-GGA GAT CTC GAT CCC GCG AAA TTA AT-3') and PER (5'-GGA GAT CTC AAA CCC CTC AAG AC-3') were complementary to upstream 5'-end of T7 promoter sequence and downstream 3'-end of T7 terminator sequence, respectively. Both primers contained the restriction enzyme *Bgl*II used to introduce the expression cassette *PlipB1T *and *PlipB3T *into the expression plasmid pELipAB^a^. The PCR products were first subcloned into the cloning vector pCR2.1 and then digested with *Bgl*II and introduced into pELipAB^a ^which was also digested with *Bgl*II resulting in pELipAB-LipB1^a ^and pELipAB-LipB3^a ^harboring two independent expression cassettes. Each expression cassette contained the complete set of the T7 promoter and T7 terminator, so that the direction of each cassette does not influence on the expression level of the other protein. The PCR mixture contained 0.5 μl DNA template (50 ng); 1.2 μl each primer (10 pmol), 10 μl PCR master mix, supplemented with 7 μl of distilled water to a final volume of 20 μl. The thermocycler conditions were performed as follows: 94°C/5'; 35 cycles of 94°C/1', 61°C/1', 72°C/30″; 72°C/10'.

### Construction of plasmids pELipA-LipB1^a ^and pELipA-LipB3^a^

The cassettes *PlipB1T *and *PlipB3T *(Figure 4), amplified from the templates pELipB1^a ^and pELipB3^a^, respectively, were inserted into the plasmid pELipA^a ^resulting in the dual expression cassette plasmids pELipA-LipB1^a ^and pELipA-LipB3^a ^harboring two expression cassettes *PlipAT *+ *PlipB1T *and *PlipAT *+ *PlipB3T*, respectively. The construction of these plasmids was similar to the construction of the dual expression cassette plasmid pELipAB-LipB1^a ^and pELipAB-LipB3^a ^described in details as above mentioned.

### Co-expression of lipase and chaperone

The transformants *E. coli *BL21 (pELipAB^a^, pELipAB-LipB1^a^, pELipAB-LipB3^a^, pELipA-LipB1^a^, pELipA-LipB3^a^) and the transformants *E. coli *BL21 (pELipAB^a ^+ pELipB1^k^, pELipAB^a ^+ pELipB1^k^, pELipA^a ^+ pELipB1^k^, pELipA^a ^+ pELipB1^k^) were cultivated in 5 ml of LB medium containing only 5 μl of ampicillin (100 mg/ml) and 5 μl of ampicillin (100 mg/ml) with 5 μl kanamycin (10 mg/ml), respectively, overnight, at 37°C with agitation at 220 rpm. Fifty μl of the overnight culture were transferred into 50 ml of LB medium containing only 50 μl of ampicillin (100 mg/ml) and 50 μl of ampicillin (100 mg/ml) with 5 μl kanamycin (10 mg/ml), respectively. The cultures were grown at 37°C on orbital shaking incubator at 220 rpm for more 3 hours until OD_600 nm _reached 0.6, then supplemented with 500 μl of IPTG (100 mM). The cultures were incubated at 37°C on orbital shaking incubator at 220 rpm for 1-4 hours of induction. Cells were harvested by centrifugation at 6000 rpm for 10 min at 4°C.

### Enzyme preparation

The cell pellet (of 250 mg cell wet weight) of 50 ml LB medium culture was harvested by centrifugation at 6000 rpm for 10 min and washed 3 times with distilled water. The cell pellet was resuspended in 1 ml of 50 mM potassium phosphate pH 8 and disintegrated by ultrasonic waves (3 × 1 min with 1 min pause). The cell lysate was centrifuged at 13000 rpm and 4°C for 15 min to separate the soluble fraction and precipitate. The pellet was resuspended in 1 ml of 50 mM potassium phosphate pH 8. The culture supernatant, cell lysate, cell lysate's supernatant (soluble fraction) and cell lysate's precipitate (inclusion bodies) were used for lipase activity estimation as well as for SDS-PAGE.

### Lipase activity estimation

Lipase activity was estimated using olive oil as substrate in a pH-stat 718 (Metrohm) as described previously [17] with some modifications. One percent (v/v) of olive oil was emulsified in distilled water containing 0.5% (w/v) of gum Arabic as stabilizer using a homogenizer (Blender, USA) for 3 × 1 min at maximum speed. A 20 ml volume of the triglyceride solution was heated to 55°C and adjusted to pH 8. Autohydrolysis was measured in 10 min without addition of enzyme. After addition of 10 μl of the lipase solution, the activity was measured with a pH-stat 718 (Metrohm AG, Herisau, Switzerland) for 10 min. One unit of lipase activity was defined as the amount of enzyme, which released 1 μmol of fatty acid per min under standard assay conditions. All measurements were carried out three times and from values the average value was taken.

### Gel electrophoresis

The expression level and molecular mass of the lipase and chaperone was determined by 12.5% SDS polyacrylamide gel electrophoresis with Biometra equipment (Göttingen, Germany) [30]. SDS-PAGE was usually performed with gels of 12.5% (w/v) of acrylamide according to the manufacturer's recommendations. An amount of 0.5 mg cell wet weight was loaded on each well. The expression level was determined by densitometry analysis Dolphin 1-D software operation (Wealtec Biosciences Co., Ltd, Taipei, Taiwan). For lipase activity staining [31], gels were incubated in 50 ml 0.1 M Tris-HCl buffer pH 7.5 containing 0.5% (w/v) of Triton X-100 for 30 min to renature the lipase. Then the gels were stained for 30 min in a mixture of 50 ml 0.1 M Tris-HCl buffer pH 7.5 containing 20 mg α-naphthyl acetate which was dissolved before in 5 ml acetone and 50 ml 0.1 M Tris-HCl buffer pH 7.5 containing 50 mg Fast Red TR.

### DNA and amino acid sequence alignment

Homologies of the DNA and amino acid sequences were determined with the program MegAlign DNAStar.

### Effect of culture factors on active lipase production

To figure out the effect of the substrate and detergents on the production of the active lipase, 0.5% (w/v) Neptune oil (substrate), gum Arabic, Tween 80 and Triton X-100 were supplemented to the LB medium. After induction by IPTG for 4 hours in different culture media supplemented without Neptune oil, with Neptune oil, gum Arabic, gum Arabic and Neptune oil, Tween 80, and Triton X-100, total proteins were applied to SDS-PAGE to estimate the expression level of the lipase in the dual expression cassette plasmid systems BL21/pELipAB-LipB1^a ^and BL21/pELipAB-LipB3^a^.

## Abbreviations

### Protein/enzyme

LipA: Lipase from *Ralstonia *sp. M1; LipB: Chaperone from *Ralstonia *sp. M1; LipB1: 56-aa truncated chaperone LipB; LipB3: 26-aa truncated chaperone LipB; LipA6xHis: muture Lipase fused with 6x histidine tag

### Gene

*lipA*: Lipase gene from *Ralstonia *sp. M1; *lipB*: Chaperone gene from *Ralstonia *sp. M1; *lipB1*: 168-nt truncated chaperone gene *lipB; lipB3*: 78-nt truncated chaperone *lipB; lipAB*: Lipase and chaperone gene cluster

### Plasmid

pELipA^a^: pET22b(+) + *lipA*, a: ampicillin resistant marker; pELipAB^a^: pET22b(+) + *lipAB*, a: ampicillin resistant marker; pELipB1^a^: pET22b(+) + *lipB1*, a: ampicillin resistant marker; pELipB3^a^: pET22b(+) + *lipB3*, a: ampicillin resistant marker; pELipB1^k^: pET28a(+) + *lipB1*, k: kanamycin resistant marker; pELipB3^k^: pET28a(+) + *lipB3*, k: kanamycin resistant marker; pELipA-LipB1^a^: pET22b(+) + *lipA *+ T7 promoter + *lipB1*+ T7 terminator, a: ampicillin resistant marker; pELipA-LipB3^a^: pET22b(+) + *lipA *+ T7 promoter + *lipB3 *+ T7 terminator, a: ampicillin resistant marker; pELipAB-LipB1^a^: pET22b(+) + *lipAB *+ T7 promoter + *lipB1 *+ T7 terminator, a: ampicillin resistant marker; pELipAB-LipB3^a^: pET22b(+) + *lipAB *+ T7 promoter + *lipB3 *+ T7 terminator, a: ampicillin resistant marker

## Competing interests

The authors declare that they have no competing interests.

## Authors' contributions

DTQ designed the experimental setup, initiated the project, assisted with data analysis and manuscript preparation, read and approved the final manuscript. HCV performed experiments of plasmid construction and expression and TTGL performed experiments of influence of additives on expression. All authors read and approved the final manuscript.
